# Supplementary material for: Intrinsic macroscale oscillatory modes driving long range functional connectivity in female rat brains detected by ultrafast fMRI
Source: Nat Commun. 2023 Feb 6;14:375. doi: 10.1038/s41467-023-36025-x (PMC9902553; doi:10.1038/s41467-023-36025-x)
Supplement: Supplementary file 3 — Description of Additional Supplementary Files [file 41467_2023_36025_MOESM3_ESM.pdf]

Description of Additional Supplementary Files:

Supplementary Movie 1 – fMRI signals in 3 rats and in 3 different conditions band-pass filtered between 0.01 and 0.3 (Sedation: medetomidine +0% isoflurane; Light anesthesia: medetomidine + 1% isoflurane; Deep anesthesia: medetomidine + 3% isoflurane). To account for expected differences in power across conditions, colorbar limits are set to ± 4 standard deviations of the band-pass filtered signals in each scan to illustrate that beyond a sharp decrease in oscillatory power, deep anesthesia is also characterized by a loss of spatial organization.

Supplementary Movie 2 - fMRI signals band-pass filtered in the frequency range that best differentiated between conditions in 3 different rats and in 3 different conditions. After removing the mean from the fMRI signals in each voxel, the signals were bandpass filtered between 0.15 and 0.25 Hz and are imaged over time. Sedation: Medetomidine only; Light Anesthesia: Medetomidine + 1% isoflurane; Deep anesthesia: Medetomidine + 3% isoflurane). To account for expected differences in power across conditions, colorbar limits are set to ± 4 standard deviations of the band-pass filtered signals in each scan.

Supplementary Movie 3 – Recorded signals are reconstructed as the linear superposition of 10 condition-specific principal components with scanspecific temporal signatures. (left) fMRI signals in N=1463 brain voxels band-pass filtered between 0.01 and 0.3 Hz recorded from a representative scan S of a rat under medetomidine only. (middle) Each of the 10 spatially defined principal modes of covariance is scaled over time by its corresponding temporal signature in scan S to illustrate the standing wave dynamics. (right) The signals recorded in scan S are reconstructed as the linear sum of the 10 principal components multiplied by their corresponding temporal signature in scan S. To account for differences in power across components, colorbar limits are set to ± 4 standard deviations of the corresponding temporal signatures.

Supplementary Movie 4 – Multi-slice acquisitions reveal that the patterns detected in the frontal slice extend to the whole brain level. fMRI signals recorded from a rat sedated with medetomidine with TR=0.350 seconds from 12 slices covering the whole brain (anterior to posterior) filtered between 0.15-0.25 Hz (no additional signal processing performed).

Source Data – Source data including the values of all the data points reported in Figure 2 b,c and Supplementary Figures 3 and 4 b,c, together with the calculation of the mean and standard error, as well as the p-values of the statistical tests reported in the plots.

Source Code – Fully commented Matlab scripts to replicate the analysis and findings reported in this manuscript, including a readme.txt file to allow quickly testing the performance of the algorithm in the full source data or in a reduced example fMRI dataset available for download in https://drive.google.com/drive/u/5/folders/1JQ_1AmP4v-HEB_ll5ZwHEaB0RtIoA17R
