# Supplementary material for: Intrinsic macroscale oscillatory modes driving long range functional connectivity in female rat brains detected by ultrafast fMRI
Source: Nat Commun. 2023 Feb 6;14:375. doi: 10.1038/s41467-023-36025-x (PMC9902553; doi:10.1038/s41467-023-36025-x)
Supplement: Supplementary file 8 — Reporting Summary [file 41467_2023_36025_MOESM8_ESM.pdf]

nature portfolio

Corresponding author(s): Noam Shemesh
Joana Cabral

Last updated by author(s): Dec 20, 2022

# Reporting Summary

Nature Portfolio wishes to improve the reproducibility of the work that we publish. This form provides structure for consistency and transparency in reporting. For further information on Nature Portfolio policies, see our Editorial Policies and the Editorial Policy Checklist.

## Statistics

For all statistical analyses, confirm that the following items are present in the figure legend, table legend, main text, or Methods section.

| n/a | Confirmed | |
|---|---|---|
| ☐ | ☒ | The exact sample size (*n*) for each experimental group/condition, given as a discrete number and unit of measurement |
| ☐ | ☒ | A statement on whether measurements were taken from distinct samples or whether the same sample was measured repeatedly |
| ☐ | ☒ | The statistical test(s) used AND whether they are one- or two-sided<br>*Only common tests should be described solely by name; describe more complex techniques in the Methods section.* |
| ☐ | ☒ | A description of all covariates tested |
| ☐ | ☒ | A description of any assumptions or corrections, such as tests of normality and adjustment for multiple comparisons |
| ☐ | ☒ | A full description of the statistical parameters including central tendency (e.g. means) or other basic estimates (e.g. regression coefficient) AND variation (e.g. standard deviation) or associated estimates of uncertainty (e.g. confidence intervals) |
| ☐ | ☒ | For null hypothesis testing, the test statistic (e.g. *F*, *t*, *r*) with confidence intervals, effect sizes, degrees of freedom and *P* value noted<br>*Give P values as exact values whenever suitable.* |
| ☒ | ☐ | For Bayesian analysis, information on the choice of priors and Markov chain Monte Carlo settings |
| ☒ | ☐ | For hierarchical and complex designs, identification of the appropriate level for tests and full reporting of outcomes |
| ☒ | ☐ | Estimates of effect sizes (e.g. Cohen's *d*, Pearson's *r*), indicating how they were calculated |

*Our web collection on statistics for biologists contains articles on many of the points above.*

## Software and code

Policy information about availability of computer code

| | |
|---|---|
| Data collection | MRI data were acquired using the software ParaVision 6.0.1. Breathing and temperature data were acquired using the PC-SAM 8.02 software from Small Animal Instruments, Inc. |
| Data analysis | All the analysis were performed on Matlab versions (R2017b and R2021b). All scripts are made available as supplementary Source Code. |

For manuscripts utilizing custom algorithms or software that are central to the research but not yet described in published literature, software must be made available to editors and reviewers. We strongly encourage code deposition in a community repository (e.g. GitHub). See the Nature Portfolio guidelines for submitting code & software for further information.

## Data

Policy information about availability of data

All manuscripts must include a data availability statement. This statement should provide the following information, where applicable:

- Accession codes, unique identifiers, or web links for publicly available datasets
- A description of any restrictions on data availability
- For clinical datasets or third party data, please ensure that the statement adheres to our policy

Data availability:
All structural and functional MRI data used in this study is available for download without restrictions in Maltab format (.mat) in:
https://drive.google.com/drive/u/5/folders/1JQ_1AmP4v-HEB_lI5ZwHEaB0RtIoA17R

## Human research participants

Policy information about studies involving human research participants and Sex and Gender in Research.

| | |
|---|---|
| Reporting on sex and gender | n/a |
| Population characteristics | n/a |
| Recruitment | n/a |
| Ethics oversight | n/a |

Note that full information on the approval of the study protocol must also be provided in the manuscript.

# Field-specific reporting

Please select the one below that is the best fit for your research. If you are not sure, read the appropriate sections before making your selection.

- [x] Life sciences
- [ ] Behavioural & social sciences
- [ ] Ecological, evolutionary & environmental sciences

For a reference copy of the document with all sections, see nature.com/documents/nr-reporting-summary-flat.pdf

# Life sciences study design

All studies must disclose on these points even when the disclosure is negative.

| | |
|---|---|
| Sample size | On a first stage, images were obtained from n=3 rats (each scanned twice in each condition) for an exploratory analysis. After the first analysis revealed statistically significant differences in band-limited spectral power between conditions surviving Bonferroni correction for multiple comparisons, we performed a replicate experiment with 3 other rats. The fact that the initial findings were replicated, increasing the statistical significance to p-values as low as 10^(-7), well beyond the Bonferroni-corrected threshold, either when considering 8 (Figure 2 and Supp.Fig S3) or 20 frequency bands (Supp. Fig. S4), confirmed that our sample size was adequate to ensure robustness of the results. |
| Data exclusions | No scan was excluded. |
| Replication | The study was first performed on 3 live rats and subsequently replicated on another 3 live rats. Differences in band-limited spectral power and in the spatial organization of the modes was detected consistently across anesthesia conditions , even at the individual rat level as shown in Supplementary figures S6 to S9. |
| Randomization | The same experimental conditions were applied to all rats. Spatial randomization was applied to the spatial pattern associated to a representative mode to demonstrate that the specific spatial organization of the phases amplifies the amplitude and spectral power of the temporal signals in Supplementary Figure S17. |
| Blinding | Blinding was not relevant as all the analyses rely on the comparison between different levels of anesthesia in rats. |

# Reporting for specific materials, systems and methods

We require information from authors about some types of materials, experimental systems and methods used in many studies. Here, indicate whether each material, system or method listed is relevant to your study. If you are not sure if a list item applies to your research, read the appropriate section before selecting a response.

## Materials & experimental systems

| n/a | Involved in the study | |
|---|---|---|
| x | | Antibodies |
| x | | Eukaryotic cell lines |
| x | | Palaeontology and archaeology |
| | x | Animals and other organisms |
| x | | Clinical data |
| x | | Dual use research of concern |

## Methods

| n/a | Involved in the study | |
|---|---|---|
| x | | ChIP-seq |
| x | | Flow cytometry |
| | x | MRI-based neuroimaging |

# Animals and other research organisms

Policy information about studies involving animals; ARRIVE guidelines recommended for reporting animal research, and Sex and Gender in Research

| | |
|---|---|
| Laboratory animals | We used 6 female Long Evans rats aged 8.3 +- 1.3 weeks. The 7th rat (Long Evans, female) used for the postmortem condition was 11 weeks old. |
| Wild animals | The study did not involve wild animals. |
| Reporting on sex | All rats were of the same gender (female) to minimize variability. The effect of gender was not addressed (but could be easily verified if necessary). |
| Field-collected samples | The study did not involve samples collected from the field. |
| Ethics oversight | All animal experiments complied with the European Directive 2010/63 (established by Portuguese law Decreto-Lei 113/2013) and followed the FELASA (Federation of European Laboratory Animal Science Associations) guidelines and recommendations concerning laboratory animal welfare. Experiments were preapproved by the Champalimaud Foundation's Internal Review Board (ORBEA) and by the Portuguese competent authority for animal welfare (DGAV, Direcção Geral de Alimentação e Veterinária) with licence number 0421/000/000/2016. |

Note that full information on the approval of the study protocol must also be provided in the manuscript.

# Magnetic resonance imaging

## Experimental design

| | |
|---|---|
| Design type | Resting-state. |
| Design specifications | Resting-state data was acquired twice for each sedation condition for each animal. Each trial was 10 min 8 s long and separated by at least 3 minutes. |
| Behavioral performance measures | N/A |

## Acquisition

| | |
|---|---|
| Imaging type(s) | Functional. |
| Field strength | 9.4 Tesla |
| Sequence & imaging parameters | Gradient-echo EPI, FOV = 21x21 mm^2, matrix size = 84 x 84, slice thickness = 1.2 mm, single coronal slice, TR = 38 ms, TE = 11 ms, Flip Angle = 15 degrees. |
| Area of acquisition | A single slice was placed between -0.2 and 1.0 mm from Bregma according to the Paxinos & Watson rat brain atlas, covering a large cortical area. |

Diffusion MRI ☐ Used ☒ Not used

## Preprocessing

| | |
|---|---|
| Preprocessing software | Matlab |
| Normalization | Data were not normalized. 2D images from the brains of different animals were aligned to the same center manually, without resizing or interpolating to keep the original fMRI signals. |
| Normalization template | No normalization template. |
| Noise and artifact removal | Given the purpose of this study, no noise or artifact removal was performed. Only mean removed and band-pass filtering. As can be seen in Supplementary Figure S5, the frequencies of heartbeat and breathing frequencies were resolved in frequency and in space at the recorded sampling rate, allowing adequate removal of high frequency components by band-pass filtering without frequency aliasing (Supplementary Figures S10 ans S11). |
| Volume censoring | As stated in the Methods section, the first 1000 volumes (out of 16000) corresponding to the first 38 seconds of recordings were discarded in each fMRI scan to remove the initial transient until stabilization of the recordings. |

## Statistical modeling & inference

| | |
|---|---|
| Model type and settings | Both covariance and Pearson's correlation were used to obtain the principal modes of functional connectivity. As shown in Supplementary Figures S12 and S13, covariance returned more modes with eigenvalues above postmortem than Pearson's correlation. |

Effect(s) tested

Rats were scanned under 3 different levels of sedatives/anaesthetics in resting condition (no stimulus) .

Specify type of analysis: ☒ Whole brain ☐ ROI-based ☐ Both

Statistic type for inference
(See Eklund et al. 2016)

The following measures were estimated for each scan in order to compare between conditions, namely: 1) Mean voxel power in each frequency band, 2) Number of covariance modes in each frequency band, 3) Peak Frequency; 4) Resonance Q-factor for each principal component.

Correction

All statistics reported were corrected for Family-Wise Error Rate using Bonferroni correction, by dividing the threshold of 0.05 by the total number of hypothesis tests performed, i.e., considering both the number of conditions (3) and frequency bands (8 in Figure 2, or 20 in Supp. Fig. S4), totalizing 24 or 60 independent comparisons, as explained in the text and figure legends.

## Models & analysis

| n/a | Involved in the study |
|---|---|
| ☐ | ☒ Functional and/or effective connectivity |
| ☒ | ☐ Graph analysis |
| ☒ | ☐ Multivariate modeling or predictive analysis |

Functional and/or effective connectivity

Covariance was used together with Pearson's correlation to estimate functional connectivity in terms of phase relationships. We tested and Covariance returned modes with stronger eigenvalues than Pearson's correlation.
